# Author Correction: Symptoms and syndromes associated with SARS-CoV-2 infection and severity in pregnant women from two community cohorts

**DOI:** 10.1038/s41598-022-10372-z

**Published:** 2022-04-12

**Authors:** Erika Molteni, Christina M. Astley, Wenjie Ma, Carole H. Sudre, Laura A. Magee, Benjamin Murray, Tove Fall, Maria F. Gomez, Neli Tsereteli, Paul W. Franks, John S. Brownstein, Richard Davies, Jonathan Wolf, Tim D. Spector, Sebastien Ourselin, Claire J. Steves, Andrew T. Chan, Marc Modat

**Affiliations:** 1 School of Biomedical Engineering and Imaging Sciences, King’s College London, 9th floor, Becket House, 1 Lambeth Palace Road, London, SE1 7EU UK; 2 Boston Children’s Hospital and Harvard Medical School, Boston, MA USA; 3 Clinical and Translational Epidemiology Unit, Massachusetts General Hospital, Boston, MA USA; 4 Department of Women and Children’s Health, School of Life Course Sciences and the Institute of Women and Children’s Health, King’s College London, London, UK; 5 Department of Medical Sciences and Science for Life Laboratory, Uppsala University, Uppsala, Sweden; 6 Department of Clinical Sciences, Lund University Diabetes Centre, Jan Waldenströms gata 35, 21428 Malmö, Sweden; 7 Zoe Global Limited, London, UK; 8 Department of Twin Research and Genetic Epidemiology, King’s College London, London, UK

Correction to: *Scientific Reports* 10.1038/s41598-021-86452-3, published online 25 March 2021

The Funding section in the original version of this Article was incomplete.

“This work was supported by Zoe Global. The Department of Twin Research receives grants from the Wellcome Trust (212904/Z/18/Z) and Medical Research Council/British Heart Foundation Ancestry and Biological Informative Markers for Stratification of Hypertension (AIMHY; MR/M016560/1), and support from the European Union, the Chronic Disease Research Foundation, Zoe Global, the NIHR Clinical Research Facility and the Biomedical Research Centre (based at Guy’s and St Thomas’ NHS Foundation Trust in partnership with King’s College London). The School of Biomedical Engineering & Imaging Science and Centre for Medical Engineering at King’s College London receive grants from the Wellcome/EPSRC Centre for Medical Engineering [WT 203148/Z/16/Z]. E.M. is funded by the ‘Skills Development Scheme’ of the Medical Research Council UK. C.M.A. is funded by NIDDK K23 DK120899 and the Boston Children’s Hospital Office of Faculty Development Career Development Award. CHS is supported by an Alzheimer's Society Junior fellowship (AS-JF-17-011). W.M., J.S.B. and A.T.C. are supported by the Massachusetts Consortium on Pathogen Readiness (MassCPR) and Mark and Lisa Schwartz. Most of the mentioned funding schemes are externally peer reviewed for scientific quality, and rely on the involvement of patient and public panels in either the design or evaluation phases, or both.”

Now reads:

“This work was supported by Zoe Global. The Department of Twin Research receives grants from the Wellcome Trust (212904/Z/18/Z) and Medical Research Council/British Heart Foundation Ancestry and Biological Informative Markers for Stratification of Hypertension (AIMHY; MR/M016560/1), and support from the European Union, the Chronic Disease Research Foundation, Zoe Global, the NIHR Clinical Research Facility and the Biomedical Research Centre (based at Guy’s and St Thomas’ NHS Foundation Trust in partnership with King’s College London). The School of Biomedical Engineering & Imaging Science and Centre for Medical Engineering at King’s College London receive grants from the Wellcome/EPSRC Centre for Medical Engineering [WT 203148/Z/16/Z]. The work performed on the Swedish part of the study is supported by grants from the Swedish Research Council, Swedish Heart-Lung Foundation and the Swedish Foundation for Strategic Research (LUDC-IRC 15-0067). E.M. is funded by the ‘Skills Development Scheme’ of the Medical Research Council UK. C.M.A. is funded by NIDDK K23 DK120899 and the Boston Children’s Hospital Office of Faculty Development Career Development Award. CHS is supported by an Alzheimer's Society Junior fellowship (AS-JF-17-011). W.M., J.S.B. and A.T.C. are supported by the Massachusetts Consortium on Pathogen Readiness (MassCPR) and Mark and Lisa Schwartz. Most of the mentioned funding schemes are externally peer reviewed for scientific quality, and rely on the involvement of patient and public panels in either the design or evaluation phases, or both.”

The original Article has been corrected.

